# But how green is it actually? Calculating the environmental footprint of kidney care environmental optimizations within haemodialysis

**DOI:** 10.1093/ckj/sfaf220

**Published:** 2025-08-11

**Authors:** Brett Duane, James Larkin, Marialuisa Caiazzo, Marta Arenas, Anita Griffin, Julia Audije-Gil, Frances Mortimer, Harriet Attwell, Aycan Yasar, Rodrigo Martínez-Cadenas, Marta Arias-Guillén, Miguel Gomez, Abass Fehintola, Ingeborg Steinbach, Alberto Ortiz

**Affiliations:** Trinity College Dublin, Dublin, Ireland; Trinity College Dublin, Dublin, Ireland; Mozarc Medical, Italy; Department of Renal Transplantation and Nephrology, Fondacion Renal; Trinity College Dublin, Dublin, Ireland; Department of Renal Transplantation and Nephrology, Fondacion Renal; Centre for Sustainable Healthcare; Centre for Sustainable Healthcare; Centre for Sustainable Healthcare; Nephrology and Hypertension Renal Unit, IIS-Fundacion Jimenez Diaz UAM, Madrid, Spain; Clinic Barcelona; Clinic Barcelona; Trinity College Dublin, Dublin, Ireland; Centre for Sustainable Healthcare; Nephrology and Hypertension Renal Unit, IIS-Fundacion Jimenez Diaz UAM, Madrid, Spain

**Keywords:** carbon footprint, kidney care, water footprint, forced labour

## Abstract

**Background:**

Environmental optimizations in kidney care have been analysed as part of the European Union co-funded KitNewCare project.

**Methods:**

Life Cycle Assessments (LCA) using Ecoinvent database and OpenLCA software quantified optimizing resource use (e.g. dialysis machines, reducing flow rates, incremental dialysis), energy-saving measures (e.g. solar energy, efficient lighting) and travel reduction (e.g. home dialysis, telemedicine). Efforts in waste management involve transitioning clinical waste to domestic waste streams, recycling and pyrolysis. Water-saving practices include reclaiming water for non-potable uses and efficient treatment systems.

**Results:**

LCA quantified these interventions, revealing significant environmental particularly in reducing travel and resource use. Travel optimizations yielded the most significant CO_2_ equivalent savings, while incremental dialysis also conserved water and reduced greenhouse gas emissions.

**Conclusion:**

The study underlines the importance of prioritizing impactful interventions to minimize the environmental footprint of chronic kidney disease care while maintaining clinical efficacy. Challenges include adapting strategies to local contexts, ensuring economic feasibility and integrating renewable energy sources based on regional energy mixes.

## INTRODUCTION

Kidney disease affects approximately 850 million people worldwide [1], with chronic kidney disease (CKD) recognized as a leading contributor to global morbidity and mortality. By 2050 CKD is projected to become one of the top 10 causes of death [2]. CKD accelerates biological aging and increases the risk of premature death. However, some patients survive to progress to kidney failure, a condition which would potentially require kidney replacement therapy (KRT) by dialysis (usually haemodialysis) or kidney transplantation. Countries that can afford to offer KRT to their population dedicate 2%–4% of the healthcare budget to treat just 0.1%–0.2% of the population with kidney failure [3]. As the global population ages, the prevalence of CKD will continue to rise, and with it, the environmental impact of KRT will increase [4], as more countries provide this level of care. At the same time, climate change poses a growing threat to human health driving urgent efforts to reduce greenhouse gas (GHG) emissions [5]. The European Commission has introduced a series of proposals aimed at aligning the European Union (EU)’s climate, energy, transport and taxation policies with the goal of cutting net GHG emissions by at least 55% by 2030, compared with 1990 levels [6]. Given the resource-intensive nature of kidney care—requiring substantial energy, water and medical supplies—the nephrology community must take proactive steps to align with these sustainability goals.

Recognizing this need, the Centre for Sustainable Healthcare (CSH) as part of their Green Nephrology Programme (started in 2009) are exploring how kidney care could reduce its environmental footprint while maintaining high-quality patient care. The CSH applies the Triple Bottom Line framework, which evaluates healthcare sustainability across three key dimensions [7]:

- People (social impact)—ensuring quality patient care, equitable access to services, employee well-being and community health outcomes.
- Planet (environmental impact)—implementing sustainable practices to reduce waste, improve energy efficiency and minimize the carbon footprint of kidney care.
- Profit (economic sustainability)—enhancing cost-effectiveness, ensuring the financial viability of kidney care services and maintaining affordability for patients. The optimizations evaluated were diverse, ranging from processes to minimise resource use (medical supplies, energy), to innovative waste disposal strategies.

Since then, additional sustainable initiatives have been developed. Some of the interventions range from a reduction in resource use (procurement, energy, waste production, travel) to a focus on education, behavioural change or comparison of resource use across sites. These interventions had been reviewed inside the KitNewCare project, an EU co-funded initiative focused on improving the environmental sustainability of kidney healthcare. The project positions kidney care—known for its high disease burden and substantial resource use—as a model to drive systemic changes towards sustainability prioritizing impactful interventions to minimize the environmental footprint of CKD care while maintaining clinical efficacy and patient safety [8].

Evaluating the environmental impact of healthcare products and processes requires a structured approach. Attributional Life Cycle Assessment (LCA) is widely used method that examines the direct environmental burdens of a product, process or system throughout its life cycle on the existing system and the direct contributions to environmental burdens. It provides a static snapshot of emissions and resource use, typically using average data rather than predicting future changes or indirect market effects [9].

In a recently published textbook on a different aspect of healthcare, we highlight that an ideal healthcare process or product should prioritize patient safety while minimizing reliance on fossil fuels. Wherever possible, the products should be sourced from renewable materials while allowing for repeated reuse or local processing. Additionally, they should be packaged sustainably or minimally, biodegrading into harmless natural components. Where feasible, the product should be easy to clean and reuse, designed for straightforward recycling, and either transported via clean energy vehicles or produced directly at the point of care. Ethical manufacturing practices and the use of renewable energy should also be fundamental to its production [10].

Water use is an environmental impact measure in LCA, similar to how carbon footprint is measured. The LCA analysis refers to the total volume of freshwater consumed directly and indirectly during the production, use and disposal of the specified items or activities. This includes water extracted for raw materials, manufacturing processes and wastewater treatment [11].

This paper was written to calculate and compare a diverse range of interventions in terms of the triple bottom line specifically looking at changes in carbon and water footprint using attributional LCA. The social impact is covered in the second paper in this series.

## METHODOLOGY

As part of the KitNewCare project, a rapid review of sustainability interventions in kidney care was conducted. The reviewed interventions were categorized into procurement and resource optimization, energy reduction, travel reduction, water conservation and waste management. The environmental impact of these interventions was quantified using LCA, assessing changes in carbon and water footprints to provide a standardized evaluation of their sustainability benefits.

### Procurement/reducing demand for procured products

Resource optimizations included managing the dialysis machine, adjusting the dialysis fluid flow (Qd) to patient body size (K16) [12], saving dialysate by automating control of flow rates [12] or increasing the treatment time/blood flow rate (rather than dialysate flow rate) [13] using incremental haemodialysis [14], lowering the dialysis fluid temperature to manage intradialytic hypotension [15], online priming to reduce need for saline bags [16], using the dialysate autoflow facility on the Fresenius 5008 machine [17], and reducing the number of disinfections of the dialysis machines to once in 24 h in a staggered manner and replacing the others with a rinsing process [18].

Centres also focused on making smaller changes, such as asking people to bring their own blankets [19], reducing food waste by asking patients to pre-order their preferred sandwiches [16] and reducing paper use through paperless haemodialysis blood result reporting [20].

### Energy

Energy reduction initiatives included heat exchangers [21], lighting upgrades [22], using solar power to assist with haemodialysis [23] and automatic IT shutdown [24].

### Travel reduction

Travel reduction initiatives included developing a renal database to allow remote monitoring of patients, increasing home haemodialysis [25, 26], managing patients in primary care to reduce travel distance [27], using remote monitoring for transplantation follow-up [28] and working [29]. The use of central delivery of acid [30] and/or more concentrated acid dialysis bags (diluted on site) also reduce transportation and therefore associated GHG emissions [31].

### Water reduction

Many renal centres used systems to reclaim reverse osmosis water [32–34] or upgraded their water treatment system [35]. Some focused on optimizing the size and location of the reverse osmosis plant [36].

### Waste management

Waste was segregated in various establishments. Domestic waste was segregated into black bags rather than all waste being discarded in clinical waste bags, as was the previous practice, and diverting bicarbonate containers from the clinical waste stream to domestic waste. Centres opted for reduction of Griff Bins [37], other centres introduced baling and recycling for their plastic and cardboard waste [38, 39], or removing their black bag waste attempted to redirect their non-contaminated dialysis waste from clinical to domestic waste.

To ensure an LCA is reproducible, it is essential to define a precise functional unit (a well-specified comparison unit), clearly state information sources, outline necessary assumptions, address ethical and data protection considerations, and establish a methodology for deriving environmental impacts from the collected data. Detailed descriptions of these elements are provided in the following subheadings.

#### Functional unit

The comparative functional unit represents a specific innovative intervention applied over 1 year, assuming three haemodialysis sessions per week allocated to one patient. In contrast, the standard functional unit is the same but corresponds to the conventional care approach.

#### Sources of information

To ensure consistency and comparability, the LCA relied on data sourced from publicly available online resources or Spanish clinical dialysis centres, as well as information gathered during the KitNewCare study [8].

### Overall assumptions

Assumptions can be seen in Table 1. To provide a more concise analysis, only a few indices were considered, such as carbon equivalent emissions (CO2e using Ecoinvent EF v3.1) and water deprivation (Ecoinvent EF v3.1). The decision on environmental aligns with KitNewCare methodology and was informed by advice from the KitNewCare stakeholder group and our external stakeholder group.

**Table 1:** 

Innovation	Functional unit	Comparative unit/standard care	Summary and assumptions
Procurement optimizations			
Using a 3 mL syringe	A 3 mL Luer lock syringe	A 10 mL Luer lock syringe	The LCA of a Luer lock syringe was assessed—70% of the syringe was estimated to calculate environment savings
Going paperless	The use of electronic resources equivalent to paper used for patient records	Six pieces of paper per patient per hospital visit used for patient records	We assumed one piece of paper was the same as 1 min use of (i) a computer and (ii) the internet. In addition, a Carnegie Mellon University study concluded that the (iii) energy cost of data transfer and storage is about 7 kWh per gigabyte
			One page is 5 kbyte, which works out at 0.00024 kWh for each piece of paper [41]
			We subtracted the environmental cost of the data storage and electronic appliance from the saved paper costs
Pre-ordering sandwiches	Providing sandwiches during patient care using an ordering process to reduce waste	Providing sandwiches during patient care without ordering	
Food choices: using vegetarian vs beef lasagne	Providing vegetarian lasagne for each patient visit	Providing beef lasagne for each patient visit	
Incremental HD	Patients start with fewer dialysis sessions per week and increase as needed, to conserve resources and reduce patient burden	Patients have usual standard of care	See Appendix 3 for more details. Only the use of the machine was measured not the build of the machine
Not using the saline bag	Not using a 1-L saline bag for every dialysis session	The use of a 1-L saline bag for every dialysis session	
Not offering blankets	The use of a 100% cotton blanket weighing 2 kg for 30% of visits	The use of a 100% cotton blanket weighing 2 kg for 100% of visits	The seed, cotton, yarn processes all taken from Ecoinvent 3.7.1. It was assumed based on Vozzola like a gown the cotton blanket would be used 60 times before discarded [42]. Calculations were made using laundry processes (transport, washing, drying) as per 3.7.1. In order to calculate the environmental impact, 70% of a blanket was calculated. The blanket was assumed to weigh 2.2 kg based on products online [43]
Energy optimizations			
Automatic IT shutdown	The use of a laptop for one patient for 1 year of HD which is always shut down when not in use	The use of a laptop for one patient for 1 year of HD which is never shut down	It was assumed that one laptop would be used for four patients. The study was based on a CSH case study [44] with 16× laptops running for 24 h and 7 days a week would (consume 3916 kWh annually); we then compared 16× laptops running 14 h a day for seven days (consume 2563 kWh annually)
Heat exchange	The use of a standard dialyser with a heat exchanger for one patient for 1 year of HD	The use of a standard dialyser without a heat exchanger for one patient for 1 year of HD	Energy savings were estimated by comparing a standard cycle on a machine with heat exchanger to one without. Results in the dialysis unit showed 5.2 kWh vs 4.7 kWh (with heat exchanger), a saving of 0.5 kWh per treatment, or approx. 10%. This was mapped for the year
Changing lightbulb	The use of a T8 lighting for one patient for 1 year of HD.	The use of a T5 lighting for one patient for 1 year of HD	Assume lighting was needed for each patient for 3 half days a week, and machine savings were calculated at 12 sessions a week (not working Sunday)
			Assume T5 are 9% more efficient [45]
Using solar energy	The energy use for one patient for 1 year of HD using roof mounted solar energy	The energy use for one patient for 1 year of HD using standard Spanish grid electricity	The energy used was derived from Arias-Guillén [46]
Lowering the dialysis fluid temperature	The energy use setting standard dialysate temperature at 36.5°C	Personalised cooler dialysate: 0.5–0.9°C below each patient's measured pre-dialysis body temperature, with a lowest recommended dialysate temperature of 35.5°C (ref)	To decrease from 36.5°C to 35.5°C save 80 Wh in a 4 h dialysis treatment, representing 2% of the total energy expenditure of the dialysis monitor [47]
Travel optimizations			
*Travel optimizations*	The mode of transport for a patient for 1 year of HD consists of 10% of patients using a minibus and 90% using a car	The distance a patient travels for 1 year of HD using a car	We know that patients travel far for their HD. We used the Irish figure of 29 km [48] one way and calculated a 10% reduction
	The mode of transport for a patient for 1 year of HD consists of 50% of patients using a minibus and 50% using a car		An average of four patients sharing a minibus when going to a HD centre
Waste optimizations			
Optimizing waste	The amount of waste for a HD patient for 1 year of HD using sophisticated pyrolysis treatment for 90% of clinical waste/10% incineration	The amount of waste for a HD patient for 1 year of HD using incineration only	Used 2.9 kg of waste per dialysis session (three times a week for 1 year of HD)
Saving water	The amount of water for a HD patient for 1 year of HD when this water is diverted for grey water uses, e.g. toilet use throughout the hospital	The amount of water for a HD patient for 1 year of HD	This does not include the environmental footprint or energy used in reprocessing the water for other uses

HD, haemodialysis.

To ensure comparability, the functional unit for each process was that used for dialysis three times a week for 1 year.

Despite most of these interventions happening in the UK, for these analyses to fit with European funding, and for consistency, we modelled the interventions as if they were happening in Spain. Spanish data (e.g. electricity use) were used where possible, to ensure that consistent comparisons could be made. The only exception was patient travel which used Irish published travel data relating to kidney care as Spanish data was unavailable [40].

The innovations are summarized in Table 1; more detail can be found the flow diagrams seen in Appendix 1 as well as the inputs for the processes in Appendix 2 (Open LCA).

#### Ethics and data protection

No personal or identifiable information was gathered from any organization or individual to calculate these footprints.

#### Data collection and analysis

The LCA methodology was applied in line with International Organisation for Standardisation standards 14 040 and European Union Product Environmental Footprint (PEF) guidance [49, 50].

In total, two separate impact environmental categories (CO2e emissions, water deprivation) were examined in this study and the Life Cycle Impact Assessment (LCIA) methods were based on PEF guidance. All inputs for each functional/comparative unit can be found in Appendix 2.

The software OpenLCA v2.11was used alongside the reference database Ecoinvent v3.7.1 for the LCIA.

## RESULTS

Small numbers less than a whole number have been rounded to ‘0’—the actual number can be seen in our data set here [51]. Contributions analysis can be seen in Appendix 4.

The results highlight the extent to which different interventions influence carbon emissions (CO2e) and water deprivation.

### Resource savings and procurement (Table 2)

The procurement-related interventions demonstrated a varied impact on environmental sustainability. The most significant reductions in water deprivation were observed in eliminating cotton blankets (116 kg CO₂, 899 m³ water). Incremental haemodialysis resulted in the highest environmental saving with an additional 316 kg of CO₂ emissions saved along with 137 m³ of water deprivation. Surprisingly, pre-ordering sandwiches saved 53 kg CO_2_ and 90 m^3^ of water. Smaller but notable improvements were seen with simple changes, such as going paperless (saving 1 kg CO₂, 0 m³ water) and eliminating saline bags (saving 3 kg CO₂, 2 m³ water).

**Table 2:** The carbon and water footprint of saving in resources.

Item	CO2e (kg)	Water deprivation (m^3^)
Procurement		
Using a 3 mL syringe	4	2
Going paperless	1	0
Pre-ordering sandwiches	53	90
Using beef vs vegetarian lasagne	19	23
Incremental haemodialysis	316	137
Not using the saline bag	*3*	*2*
Not offering cotton blankets	116	899

a A negative score implies a worsening rather than an improvement.

### Energy savings (Table 3)

Energy conservation strategies showed varied potential in reducing environmental footprints. The greatest impact was achieved using solar energy, which saved 646 kg of CO₂ emissions and 52 m³ of water. Other interventions, such as heat exchange (savings of 31 kg CO₂, 9 m³ water) and automatic IT shutdown (saving 8 kg CO₂, 2 m³ water), contributed to moderate reductions, while switching lightbulbs had little environmental effect.

**Table 3:** The carbon and water footprint of saving in energy.

Energy		
Item	CO2e (kg)	Water deprivation (m^3^)
Automatic IT shutdown	8	2
Heat exchange	31	9
Changing lightbulbs	Neg	Neg
Using solar energy	646	52

### Reducing patient travel (Table 4)

Optimizing patient travel demonstrated a significant opportunity to reduce emissions and water usage. A 10% reduction in travel resulted in 282 kg of CO₂ savings and 34 m³ of water. More substantial savings were achieved with greater reductions in travel, with a 50% reduction yielding 1411 kg CO₂ and 170 m³ water saved.

**Table 4:** The environmental and water footprint of saving in patient travel.

Travel		
Item	CO2e (kg)	Water use (m^3^)
Travel optimizations 10%	175	32
Travel optimizations 50%	710	129

### Waste reduction strategies (Table 5)

Waste management practices also played a role in reducing environmental impact. Pyrolysis of waste resulted in a 735 kg reduction in CO₂ emissions and 71 m³ of water savings.

**Table 5:** The carbon and water footprint of saving in waste.

Waste item	CO2e (kg)	Water deprivation (m^3^)
Optimising waste (optimal waste e.g. pyrolysis of medical waste)	735	71

### Water conservation (Table 6)

Direct efforts to reduce water usage contributed modest but meaningful reductions in environmental impact. Water-saving interventions resulted in a total reduction of 16 kg CO₂ and 19 m³ of water saved.

**Table 6:** The carbon and water footprint of saving in water.

	CO2e (kg)	Water deprivation (m^3^)
Saving water	16	19

## DISCUSSION

Initiatives like KitNewCare project aim to address the sustainability of kidney care, serving as a model for reducing the carbon footprint of healthcare. It is important to weigh the impact of optimizations against each other to inform sustainability efforts and help clinical sites to prioritize their implementation. Numerous innovations are occurring in this field, their impacts vary across the two chosen measurements for environmental footprint (CO2e, water) The social impact of these interventions will be discussed in a later paper.

LCA involve various assumptions for much of the resource use. To ensure consistency, activity data from a renal centre in Spain were used whenever possible. However, travel patterns may differ in urban areas, especially where individuals have better access to public transport. Energy savings can also vary significantly. Where the energy mix of an EU country is high in coal and other fossil fuels, the energy savings stated here could be substantially higher. Conversely, in countries who already have a high proportion of renewable energy, installing solar panels may result in lower GHG emissions savings associated with electricity use.

For reference regarding the figures below, the average carbon footprint of a person in Europe is 5.66 tons, or 5660 kg [52], an average month's carbon footprint is around 471 kg; and the carbon footprint of driving 100 km in a large car is around 25 kg [53]. An average person uses 0.2–0.4 m^3^ every day [54].

Patient travel-related interventions demonstrated the highest savings in GHG emissions, ranging from 175 kg (10% using minibus instead of car) to 710 kg (50% using minibus instead of car). These GHG emissions primarily stem from the maintenance and distance the car travels, rather than the car's production. We did not measure changes in staff travel although we assume that similar (proportional) changes will happen with a staff travel intervention.

Water savings of between 32 and 129 m^3^ also resulted from significant changes in travel. However, unlike CO2e emissions, this water consumption is primarily associated with the production of the car's components, rather than the distance travelled or maintenance.

Ecoinvent allocates 8 g of car production for every km that the car is driven [8]. It could be argued that this allocation is only accurate for an ‘average car’ that drives a specific number of kilometres before being redundant. To reduce the amount of carbon emissions associated with car travel, it would be necessary to reduce car ownership.

Within this study, we calculated a 10% change and 50% change in patients using a shared minibus instead of car. These choices of a 10% and 50% reduction were arbitrary and solely related to plausible reductions in patient travel. Moving to a green energy powered public transport only system, e.g. through government policy, etc., might be feasible.

Travel can be reduced through various means, including increased use of public transportation, and the provision of home-based care. Home-based dialysis could be one solution. It may also be possible to allocate the patient to a closer facility, reducing travel distance. By increasing the availability of home dialysis, implementing telemedicine where clinically appropriate or coordinating ambulance services, centres have successfully reduced the number of trips patients need to make. Reducing patient travel in this respect not only decreases carbon emissions but also enhances patient convenience and access to care.

Another significant area of focus is water management. Improving the efficiency of dialysate concentrate delivery systems can contribute to overall sustainability in dialysis care. Zawierucha *et al*. highlight that liquid dialysate concentrates, commonly delivered in 10-L canisters, have a higher environmental impact due to increased transportation emissions and packaging waste [55]. In contrast, powder and semi-dry concentrates significantly reduce carbon emissions, storage space requirements and transport-related environmental burdens. The study also emphasizes the benefits of in-centre mixing systems, such as EcoMix (B. Braun) and Granumix Plus (Fresenius Medical Care), which enable the on-site production of dialysate. These systems lower packaging waste, minimize transport emissions and enhance cost-efficiency, while also reducing the risk of preparation errors. Shifting towards these more sustainable options could be a key strategy in reducing the environmental footprint of dialysis centres.

We also know from a recent Spanish paper that both water and energy consumption were lower per patient in larger centres compared with smaller ones, and in those operating daily versus those with a thrice-weekly schedule [56]. These factors had a notable impact on water usage. Despite conducting fewer dialysis sessions annually, smaller centres and those with a thrice-weekly schedule exhibited proportionally higher water and energy consumption per session. This is because the water treatment system consumes energy and water during start-up, priming, rinsing and disinfection processes, irrespective of the number of sessions conducted. Dialysis requires large volumes of water, and many centres have developed systems to reclaim and reuse water, either within the dialysis process itself or for non-potable uses such as via hospital greywater systems. These innovations can reduce the environmental burden associated with water use and lower operating costs for healthcare facilities [57]. Interestingly, water management (tap water production and associated sewerage) also has relatively high CO2e emissions, primarily due to the construction and maintenance of water production and sewerage plants. Sustainability innovators should consider the strong correlation between water use and carbon emissions [58].

Waste management strategies have also been explored to mitigate the environmental impact of dialysis. Waste management in renal centres can be expensive with the incineration costs of healthcare waste in the UK at £337 per ton (in 2018), which equates to £437 (today) when adjusted for inflation using the Bank of England calculator, yet many centres do little to manage their waste appropriately [59]. Changes in how waste is categorized, such as diverting certain materials from clinical waste streams to domestic waste, have reduced the environmental load. In addition, there have been attempts to reduce waste at the source by rethinking supply chain logistics and the materials used in dialysis care, such as switching from single-use to reusable or recyclable materials where possible. Some centres have moved away from the traditional practice of sending all dialysis waste to be incinerated, instead focusing on segregating recyclable materials and using pyrolysis or other advanced waste treatment technologies to convert clinical to domestic waste [60]. Despite offering significant planetary and health benefits, this technology is not widely implemented in Europe.

The impact of changes in resource use varied from negligible (avoiding saline bags e.g. close to 0 kg CO2e, going paperless) to moderate (altering meat types, pre-ordering sandwiches to minimize waste) to significant (not offering blankets, e.g., 116 kg CO2e, and implementing incremental dialysis).

Cotton blankets, like most textiles, are well known for their high environmental footprints [61] with textiles worldwide accounting for approximately 20% of global water production.

The current paradigm is to start and continue thrice weekly haemodialysis once kidney replacement therapy is deemed necessary. However, when dialysis is first started, there is often residual kidney function that contributes positively to patients’ wellbeing but is progressively lost over time. The blood pressure changes that occur with haemodialysis can accelerate the loss of residual kidney function; thus, it has been suggested that dialysis can be initiated incrementally, starting with once or twice weekly haemodialysis, increasing as needed to achieve adequate clearance. Incremental dialysis showed a significant reduction in CO2e, water use and frequency of forced labour. Likewise, observational studies suggest that this approach is safe and may better preserve kidney function, but clinical trials are ongoing [62–64].

GHG emission results associated with energy use revealed minimal savings from altering lighting within the nephrology organization. Automatic IT shutdown and heat exchange yielded some environmental benefits.

Another saving, albeit modest, was made using heat exchangers. Since dialysis fluid interacts with blood in the extracorporeal circuit, it must be heated to a temperature close to body temperature. Conventional systems use energy for this purpose. Heat exchangers utilize the residual heat in drained dialysate to pre-heat fresh fluid, thereby reducing the energy required to heat fluid to body temperature. It was estimated that this may reduce energy consumption for heating fluid up to 17% [65]. Savings of 31 kg CO_2_ emissions and 9 m^3^ were saved using these techniques per patient treatment per year.

The main GHG emissions savings associated with energy use were achieved by installing solar panels to meet all the energy needs of the unit. Installing solar energy is cost-effective and offers a straightforward way for some nephrology units to reduce their environmental footprint. However readers should also consider their country's energy mix before making this decision. If a country is moving towards a low carbon energy mix, the level of reduction in CO2e will not be as high.

Transitioning to vegetarian meals can substantially reduce the environmental footprint of healthcare facilities. Meat production, particularly beef, is a significant contributor to environmental deterioration. Livestock production systems contribute significantly to environmental impact globally, with meat consumption projected to increase alongside the population. By substituting meat based meals with plant based alternatives, healthcare facilities can achieve notable reductions in carbon emissions, estimated to reduce the carbon footprint of a meal by up to 50% [66].

## CONCLUSIONS

This study highlights the most effective strategies for reducing the environmental impact of kidney care. Among procurement-related interventions, incremental haemodialysis had the greatest impact, significantly reducing both CO₂ emissions and water use. Eliminating cotton blankets and pre-ordering meals also led to substantial savings. In energy conservation, solar power was the most effective intervention, while reducing patient travel—even by 10%—demonstrated a major opportunity for environmental benefit. Waste reduction and water conservation strategies contributed to smaller but meaningful improvements. The KitNewCare study provides data-driven insights to help sustainability programs prioritize high-impact interventions. As CKD prevalence rises, ongoing innovation and policy changes will be key to reducing the environmental footprint of kidney care. By focusing on the most effective solutions, healthcare systems can integrate sustainability without compromising patient care.

## Supplementary Material

sfaf220_Supplemental_File

## Figures and Tables

**Figure:**
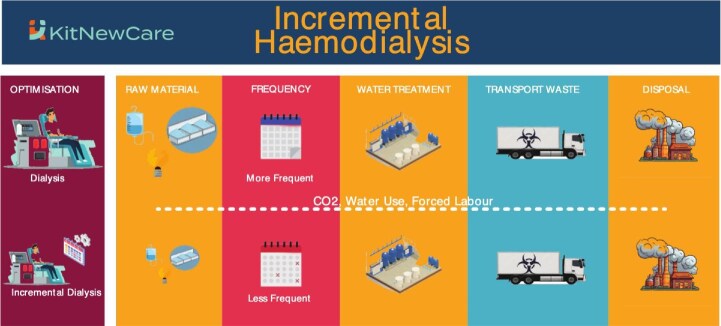


**Figure:**
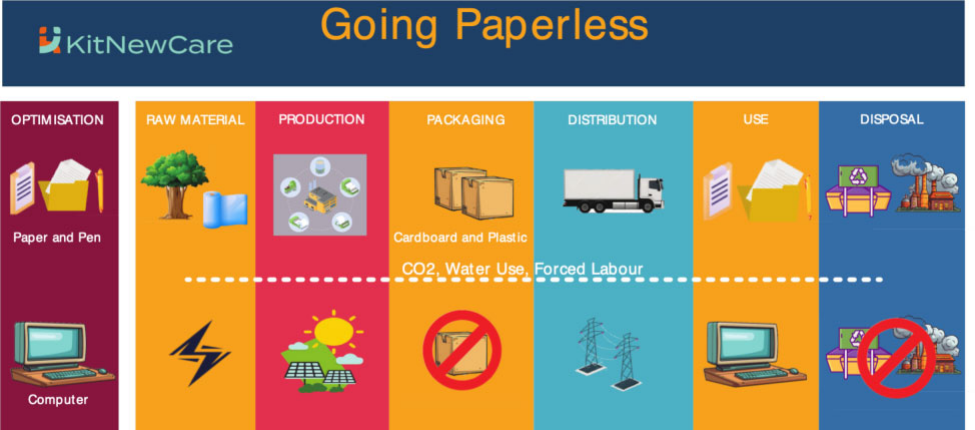


**Figure 1:**
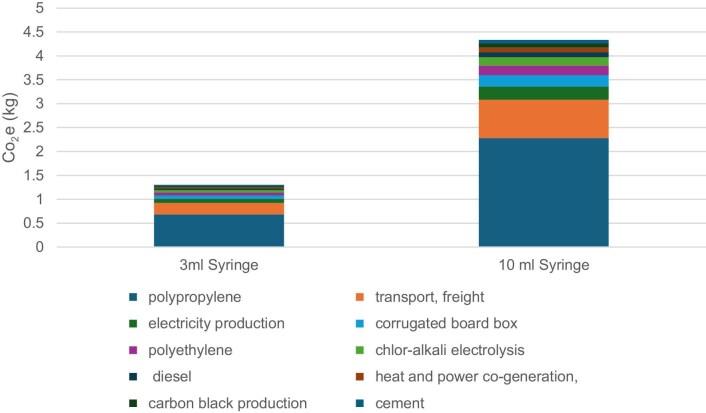
CO₂e from a 3 mL vs 10 mL syringe.

**Figure 2:**
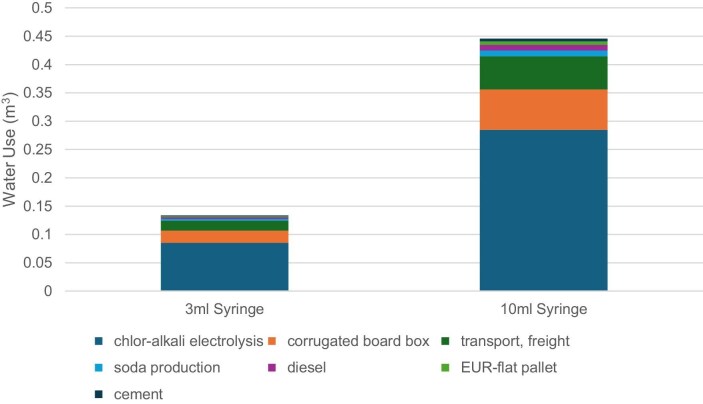
Water use from a 3 mL vs 10 mL syringe.

**Figure 3:**
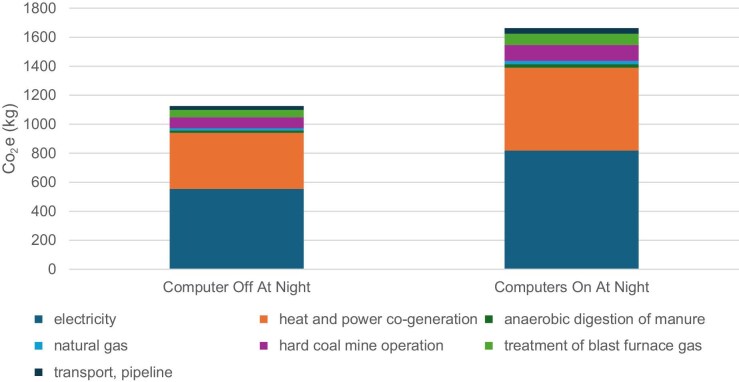
CO_2_e from a 3 mL vs 10 mL syringe.

**Figure 4:**
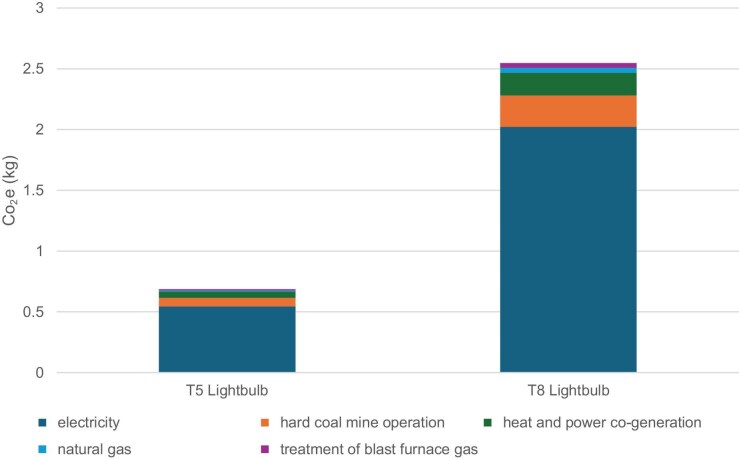
CO_2_e from changing a lightbulb from T5 to T8.

**Figure 5:**
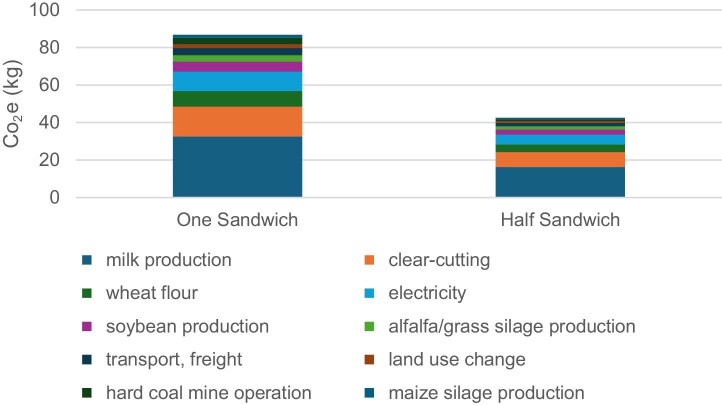
CO_2_e from saving a sandwich.

**Figure 6:**
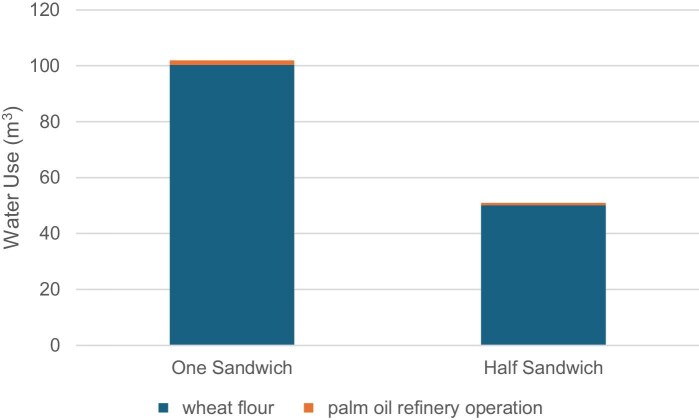
Water use from saving a sandwich.

**Figure 7:**
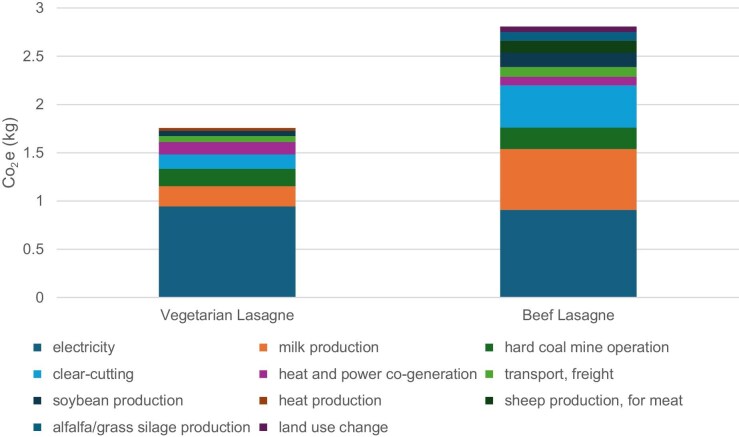
CO_2_e from beef vs vegetarian lasagne.

**Figure 8:**
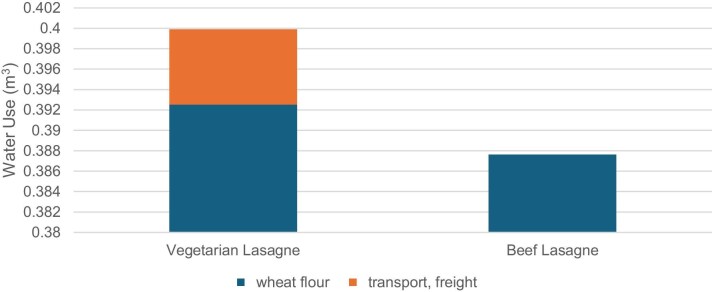
Water use from beef vs vegetarian lasagne.

**Figure 9:**
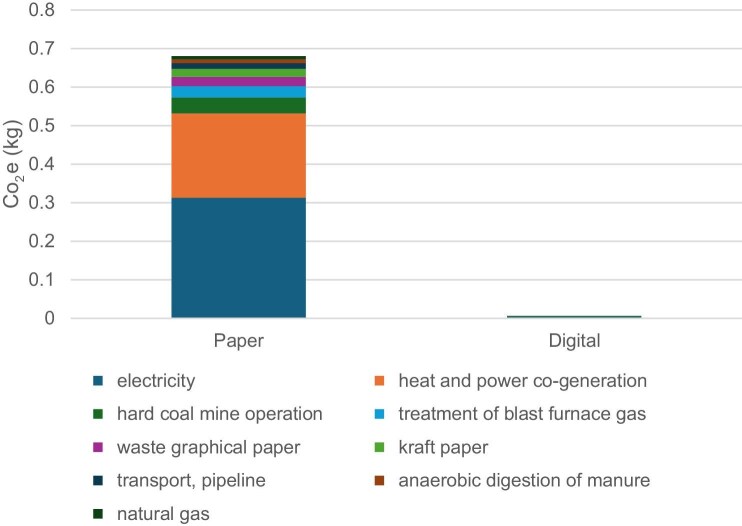
CO_2_e from going paperless.

**Figure 10:**
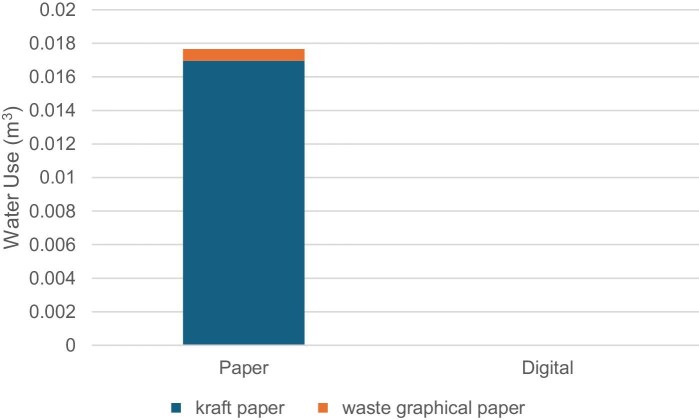
Water use from going paperless.

**Figure 11:**
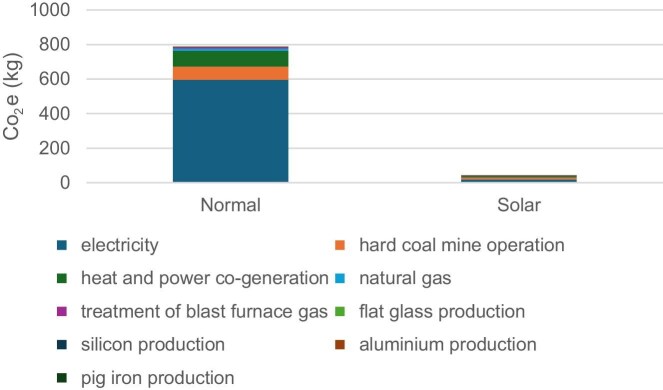
CO_2_e from using solar energy.

**Figure 12:**
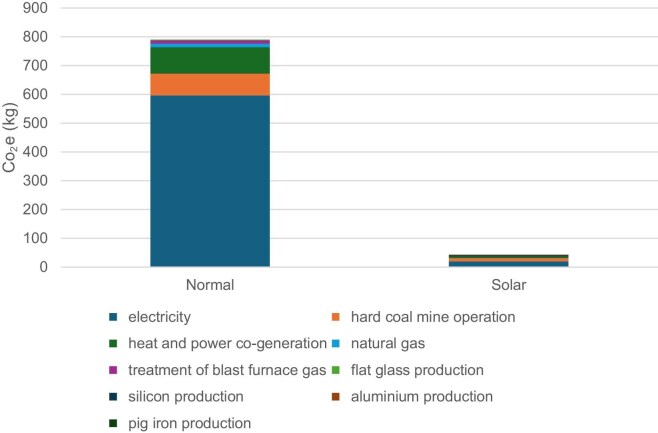
CO_2_e from saving the saline bag.

**Figure 13:**
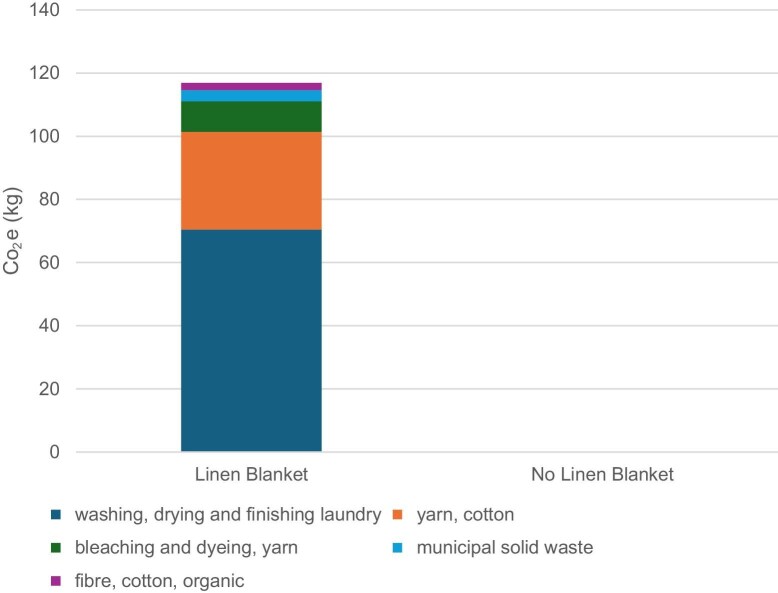
CO_2_e from not offering linen blanket.

**Figure 14:**
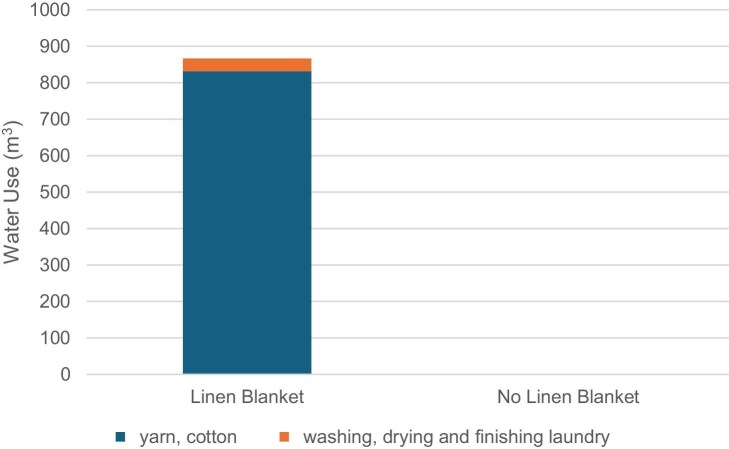
Water use from not offering linen blanket.

**Figure 15:**
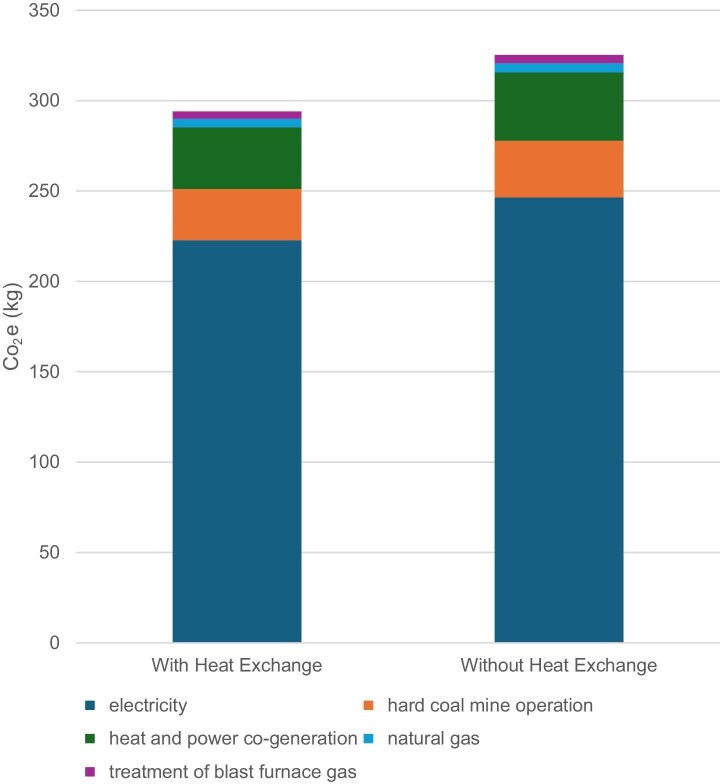
CO_2_e from heat exchange.

**Figure 16:**
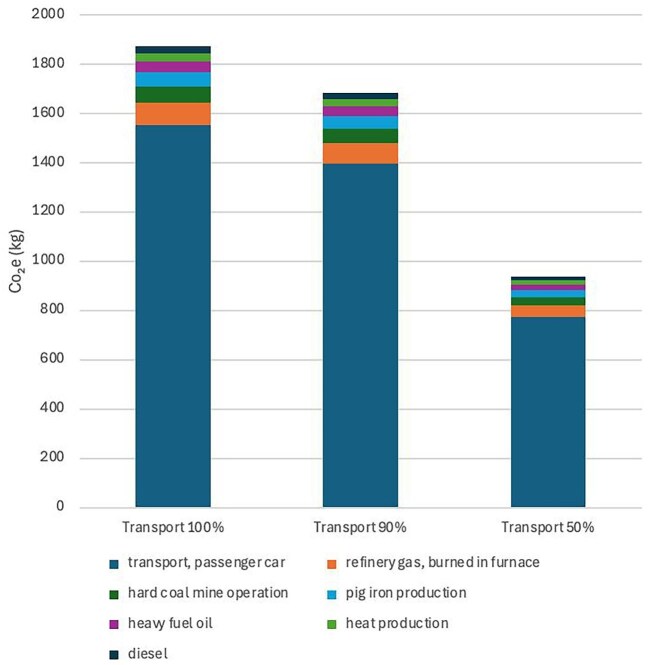
CO_2_e from travel optimizations.

**Figure 17:**
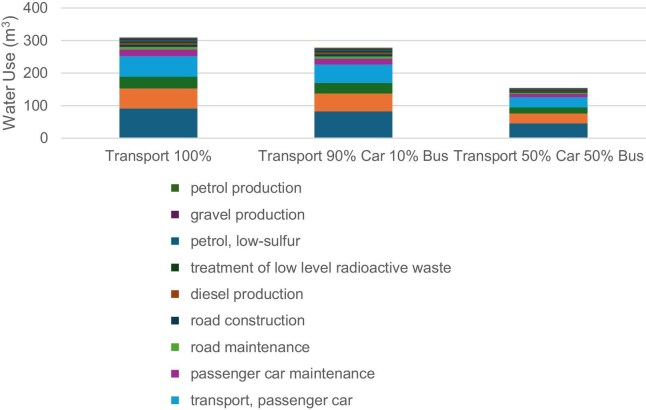
Water use from travel optimizations.

**Figure 18:**
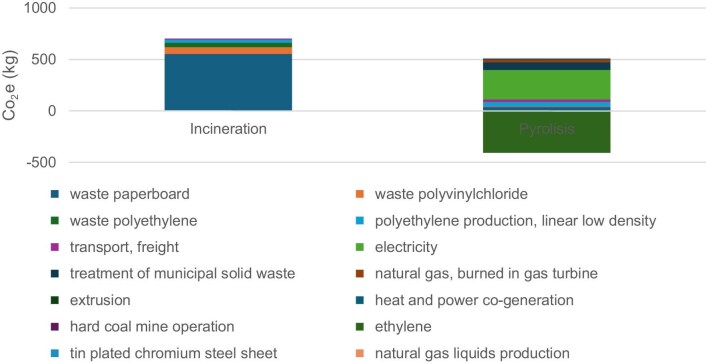
CO_2_e from waste.

**Figure 19:**
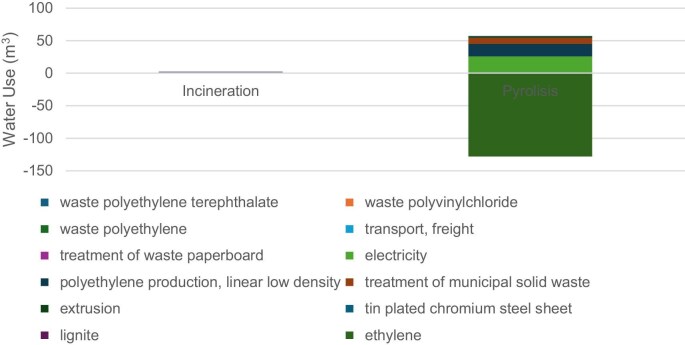
Water use from waste.

**Figure 20:**
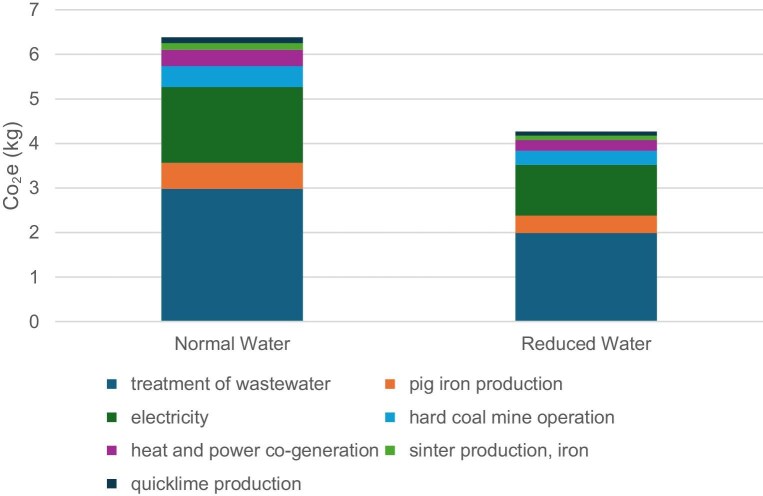
CO_2_e from saving water.

**Figure 21:**
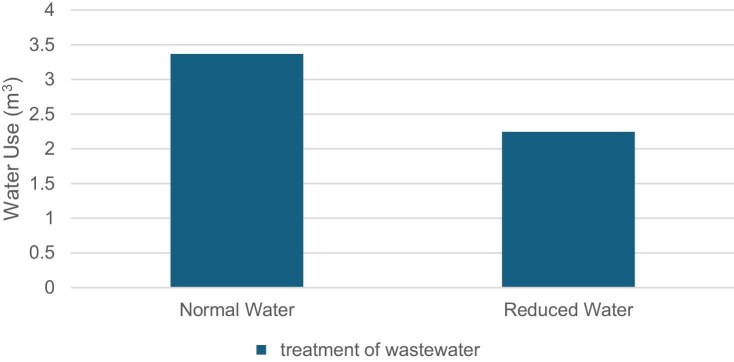
Water use from saving water.

**Figure 22:**
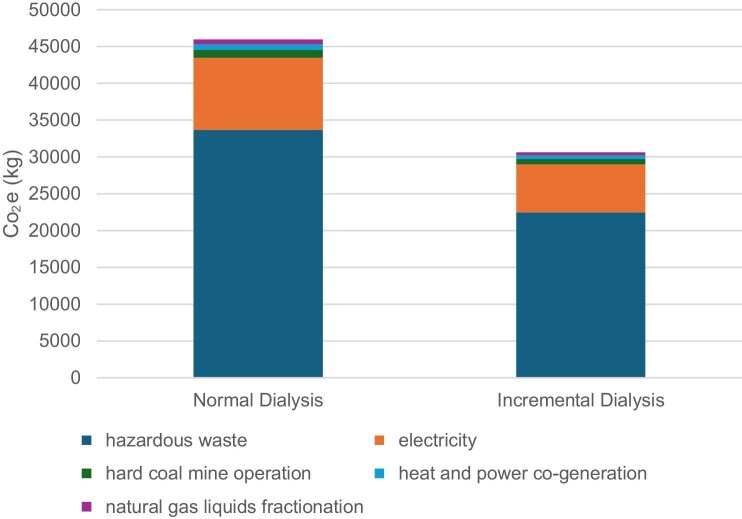
CO_2_e from incremental haemodialysis.

**Figure 23:**
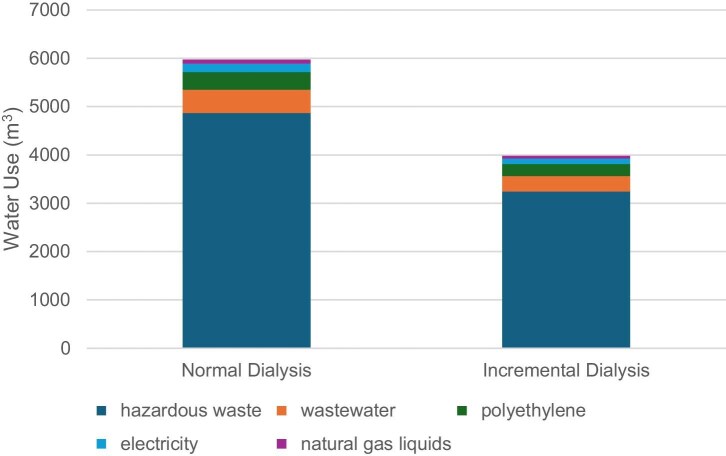
Water use from incremental haemodialysis.

## Data Availability

The data underlying this article are available in the article and in its online supplementary material.

## Notes

*Incremental haemodialysis*

*Going paperless*

## APPENDICES

### Appendix 1: Flow diagrams

*Waste optimization*

*Beef vs vegetarian lasagne*

*Pre-ordering sandwiches*

*Changing lightbulbs*

*Travel optimizations*

*Not offering linen blanket*

*Automatic IT shutdown*

*Heat exchange*

*Saving the saline bag*

*Solar assisted haemodialysis*

*Three mL versus 10 mL syringe*

### Appendix 2: Open lca inputs and outputs

Duane B, Larkin J, Fehintola A *et al.* Life Cycle Assessment Dataset for Kidney Care Environmental Optimisations within Haemodialysis [Data set]. 2024. Brett Duane. Available at: https://doi.org/10.5281/zenodo.14268014.

### Appendix 3: Information for incremental haemodialysis calculations

A comparison was made from standard 3× week to 1× or 2× week incremental schedule.

Scenario 1 refers to 51.3 weeks in 1× then moved 39 weeks to 2×.

Scenario 2 considers that 33% of patients already start with 2×.

**Table:**

Regime	Monthly sessions	Sessions	Water consumed (L)	Energy consumed (kWh)	Waste generation (kg) (modena waste mix)
3X	13	272	108 888	952.77	544.44
2X	9	188	75 384	659.61	376.92
1X	5	105	41 880	366.45	209.4
Scenario 1		152	60 800	532	304
Scenario 2		195	78 000	682.5	390
Difference		=43	=17 200	=150.5	=86
		Estimated saving:			
		From 3× to 1×	62%		
		From 3× to 2×	31%		
		Scenario 1	44%		
		Scenario 2	28%		

### Appendix 4: Results by contribution (one patient dialyzed 1 year 3×/week for 52 weeks)

Figure 1: CO_2_e from a 3 mL vs 10 mL syringe… 00

Figure 2: Water use from a 3 mL vs 10 mL syringe… 00

Figure 3: CO_2_e from a 3 mL vs 10 mL syringe… 00

Figure 4: CO_2_e from changing a lightbulb from T5 to T8… 00

Figure 5: CO_2_e from saving a sandwich… 00

Figure 6: Water use from saving a sandwich… 00

Figure 7: CO_2_e from beef vs vegetarian lasagne… 00

Figure 8: Water use from beef vs vegetarian lasagne… 00

Figure 9: CO_2_e from going paperless… 00

Figure 10: Water use from going paperless… 00

Figure 11: CO_2_e from using solar energy… 00

Figure 12: CO_2_e from saving the saline bag… 00

Figure 13: CO_2_e from not offering linen blanket… 00

Figure 14: Water use from not offering linen blanket… 00

Figure 15: CO_2_e from heat exchange… 00

Figure 16: CO_2_e from travel optimizations… 00

Figure 17: CO_2_e from waste… 00

Figure 18: Water use from waste… 00

Figure 19: CO_2_e from saving water… 00

Figure 20: Water use from saving water… 00

Figure 21: CO_2_e from incremental haemodialysis… 00

Figure 22: Water use from incremental haemodialysis… 00

*Using a 3 mL syringe*

*Automatic IT shutdown*

*Changing lightbulb*

*Pre-ordering sandwiches*

*Using beef versus vegetarian lasagne*

*Going paperless*

*Using solar energy*

*Not using the saline bag*

*Not offering linen blanket*

*Heat exchange*

*Travel optimizations*

*Waste*

*Saving water*

*Incremental haemodialysis*
