# Correction to: But how green is it actually? Calculating the environmental footprint of kidney care environmental optimizations within haemodialysis

**DOI:** 10.1093/ckj/sfag126

**Published:** 2026-05-13

This is a correction to: Brett Duane, James Larkin, Marialuisa Caiazzo, Marta Arenas, Anita Griffin, Julia Audije-Gil, Frances Mortimer, Harriet Attwell, Aycan Yasar, Rodrigo Martínez-Cadenas, Marta Arias-Guillén, Miguel Gomez, Abass Fehintola, Ingeborg Steinbach, Alberto Ortiz, But how green is it actually? Calculating the environmental footprint of kidney care environmental optimizations within haemodialysis, *Clinical Kidney Journal*, Volume 18, Issue 9, September 2025, sfaf220, https://doi.org/10.1093/ckj/sfaf220

The name of the sixth author has been emended to read “Julia Audije-Gil”.

