# Supplementary material for: But how green is it actually? Calculating the environmental footprint of kidney care environmental optimizations within haemodialysis
Source: Clin Kidney J. 2025 Aug 11;18(9):sfaf220. doi: 10.1093/ckj/sfaf220 (PMC12477476; doi:10.1093/ckj/sfaf220)
Supplement: sfaf220_Supplemental_File [file sfaf220_supplemental_file.docx]

## Appendices:

### Appendix 1: Flow diagrams:

#### Incremental haemodialysis

#### Going paperless

#### Waste Optimisation

#### Beef vs veg lasagne

#### Pre-ordering sandwiches

#### Changing Lightbulbs

#### Travel Optimisations

#### Not offering linen blanket

Automatic IT Shutdown

#### Heat Exchange

#### Saving the saline bag

#### Solar Assisted Haemodialysis

#### Three ml versus Ten ml syringe

### Appendix 2: Open LCA Inputs and Outputs

Duane, B., Larkin, J., Fehintola, A., & Martinez, R. (2024). Life Cycle Assessment Dataset for Kidney Care Environmental Optimisations within Haemodialysis [Data set]. Brett Duane. <https://doi.org/10.5281/zenodo.14268014>

### Appendix 3: Information for Incremental haemodialysis calculations

A comparison was made from standard 3x week to 1x or 2x week incremental schedule.

Scenario 1 refers to 51.3 weeks in 1x then moved 39 weeks to 2x.

Scenario 2 considers that 33% of patients already start with 2x

| Regime | Monthly sessions | Sessions | Water consumed (L) | Energy consumed (kWh) | Waste generation (Kg) (modena waste mix) |
| --- | --- | --- | --- | --- | --- |
| 3X | 13 | 272 | 108888 | 952,77 | 544,44 |
| 2X | 9 | 188 | 75384 | 659,61 | 376,92 |
| 1X | 5 | 105 | 41880 | 366,45 | 209,4 |
| Scenario 1 |  | 152 | 60800 | 532 | 304 |
| Scenario 2 |  | 195 | 78000 | 682,5 | 390 |
| **Difference** |  | **=43** | **=17200** | **=150,5** | **=86** |
|  |  | Estimated saving |  |  |  |
|  |  | From 3X to 1X | 62% |  |  |
|  |  | From 3X to 2X | 31% |  |  |
|  |  | Scenario 1 | 44% |  |  |
|  |  | Scenario 2 | 28%% |  |  |

### Appendix 4: Results by contribution (one patient dialyzed one year 3x/week for 52 weeks.)

[Figure 1: CO₂e from a 3ml vs 10ml Syringe 7](#_Toc205277685)

[Figure 2: Water Use from a 3ml vs 10ml Syringe 7](#_Toc205277686)

[Figure 3: CO₂e from a 3ml vs 10ml Syringe 8](#_Toc205277687)

[Figure 4: CO₂e from Changing a Lightbulb from T5 to T8 8](#_Toc205277688)

[Figure 5: CO₂e from Saving a Sandwich 9](#_Toc205277689)

[Figure 6: Water Use from Saving a Sandwich 9](#_Toc205277690)

[Figure 7: CO₂e from Beef vs Vegetarian Lasagne 10](#_Toc205277691)

[Figure 8: Water Use from Beef vs Vegetarian Lasagne 10](#_Toc205277692)

[Figure 9: CO₂e from Going Paperless 11](#_Toc205277693)

[Figure 10: Water Use from Going Paperless 11](#_Toc205277694)

[Figure 11: CO₂e from Using Solar Energy 12](#_Toc205277695)

[Figure 12: CO₂e from Saving the Saline Bag 12](#_Toc205277696)

[Figure 13: CO₂e from Not Offering Linen Blanket 13](#_Toc205277697)

[Figure 14: Water Use from Not Offering Linen Blanket 13](#_Toc205277698)

[Figure 15: CO₂e from Heat Exchange 14](#_Toc205277699)

[Figure 16: CO₂e from Travel Optimisations 15](#_Toc205277700)

[Figure 17 Water Use from Travel Optimisations 15](#_Toc205277701)

[Figure 18: CO₂e from Waste 16](#_Toc205277702)

[Figure 19: Water Use from Waste 16](#_Toc205277703)

[Figure 20: CO₂e from Saving Water 17](#_Toc205277704)

[Figure 21: Water Use from Saving Water 17](#_Toc205277705)

[Figure 22: CO₂e from Incremental Haemodialysis 18](#_Toc205277706)

[Figure 23: Water Use from Incremental Haemodialysis 18](#_Toc205277707)

#### Using a 3ml Syringe

Figure 1: CO₂e from a 3ml vs 10ml Syringe

Figure 2: Water Use from a 3ml vs 10ml Syringe

#### Automatic IT Shutdown

Figure 3: CO₂e from a 3ml vs 10ml Syringe

#### Changing Lightbulb

Figure 4: CO₂e from Changing a Lightbulb from T5 to T8

#### Pre-ordering sandwiches

Figure 5: CO₂e from Saving a Sandwich

Figure 6: Water Use from Saving a Sandwich

#### Using beef versus vegetarian lasagne

Figure 7: CO₂e from Beef vs Vegetarian Lasagne

Figure 8: Water Use from Beef vs Vegetarian Lasagne

#### Going Paperless

Figure 9: CO₂e from Going Paperless

Figure 10: Water Use from Going Paperless

#### Using Solar energy

Figure 11: CO₂e from Using Solar Energy

#### Not using the saline bag

Figure 12: CO₂e from Saving the Saline Bag

#### Not Offering Linen Blanket

Figure 13: CO₂e from Not Offering Linen Blanket

Figure 14: Water Use from Not Offering Linen Blanket

#### Heat Exchange

Figure 15: CO₂e from Heat Exchange

#### Travel Optimisations

Figure 16: CO₂e from Travel Optimisations

Figure 17 Water Use from Travel Optimisations

#### Waste

Figure 18: CO₂e from Waste

Figure 19: Water Use from Waste

#### Saving Water

Figure 20: CO₂e from Saving Water

Figure 21: Water Use from Saving Water

#### Incremental Haemodialysis

Figure 22: CO₂e from Incremental Haemodialysis

Figure 23: Water Use from Incremental Haemodialysis
